# Continuing medical education: SSRIs and pregnancy

**DOI:** 10.4103/0019-5545.58906

**Published:** 2010

**Authors:** Chittaranjan Andrade

**Affiliations:** Department of Psychopharmacology, National Institute of Mental Health and Neurosciences, Bangalore 560 029, India

## CME QUESTIONS

In euthymic women with a history of major depression, the planned, voluntary withdrawal of antidepressant medication prior to conception (as compared with the continuation of medication all through pregnancy) is associated with a five-fold increase in the risk of relapse during pregnancy. A longer duration of illness and a larger number of past depressive episodes each predict an increased risk of relapse. About half of the relapses occur during the first trimester.[1]

Untreated depression during pregnancy is itself associated with a large number of adverse maternal and fetal outcomes. These include an increased risk of maternal mortality,[2] poor weight gain, alcohol or illicit drug use, toxemias of pregnancy, and poor mother-child bonding[3,4] and an increased risk of pre-term delivery, low birth weight, operative delivery, and neonatal ICU admission.[5–7]

There is now an increasing trend to prescribe selective serotonin reuptake inhibitors (SSRIs) to treat depression during pregnancy; in fact, SSRIs are now first-line antidepressants during pregnancy in Denmark and many other European countries.[8,9] The epidemiology of SSRI and antidepressant use during pregnancy has been described by Tuccori *et al*.[10]

With this background, mark *True* or *False* against each of the statements in sets A and B:

### A) Teratogenicity of SSRIs after first-trimester exposure:

1. SSRIs increase the overall risk of major congenital malformations
2. SSRIs increase the overall risk of minor congenital malformations
3. SSRIs increase the risk of septal heart defects
4. SSRIs increase the risk of certain specific but rare congenital malformations

### B) Other pregnancy outcomes after exposure to SSRIs

1. SSRIs increase the risk of spontaneous abortions
2. SSRIs increase the risk of preterm delivery
3. SSRIs are associated with low Apgar scores after birth
4. SSRIs are associated with an approximately 500 g decrease in mean birth weight
5. SSRIs are associated with decreased head circumference in the newborn
6. SSRIs increase the risk of persistent pulmonary hypertension in the newborn
7. SSRI exposure during late pregnancy is associated with a neonatal SSRI withdrawal syndrome

### C) SSRI-exposed vs. SSRI-unexposed pregnancies in depressed women

1. It is better to treat depression during pregnancy than to leave it untreated because neonatal outcomes in SSRI-treated depressed women are better than those in untreated depressed women.

## CME ANSWERS

### A) Teratogenicity of SSRIs after first-trimester exposure:

1. False; 2. False; 3. True. 4. True

Pedersen *et al*,[11] describe a large, population-based, retrospective cohort study of the teratogenicity of SSRIs using data extracted from four nationwide registers in Denmark. Major and minor congenital malformations were studied in live born children in whom these malformations were detected within a year of birth. Data were excluded if women had used non-SSRI psychotropic drugs or drugs for a variety of medical indications.

There were 1370 infants who had been exposed to SSRIs and 493,113 infants who had not been exposed. In this connection, exposure was defined as the redemption of at least two prescriptions for SSRIs from 28 days before conception to 112 days after conception; the assumption was that filling repeated prescriptions implied that the women were actually taking their medications. The risks of teratogenicity with SSRIs were calculated after adjusting for maternal age, marital status, income, smoking, and other variables. This is because women who used SSRIs were more likely to have risk factors such as greater age, living alone, unmarried status, smoking etc.

Pedersen *et al*,[11] found that the risk of major malformations in SSRI-exposed vs. unexposed infants was 4.0% vs. 3.1%. The difference was not significant (adjusted odds ratio [OR], 1.21; 95% confidence intervals [CI], 0.91-1.62). No individual SSRI was associated with an increased risk. The risk of minor malformations in SSRI-exposed vs. unexposed infants was 2.8% vs. 1.5%. This difference was also not significant (adjusted OR, 0.88; 95% CI, 0.54-1.41). Again, no individual SSRI was associated with an increased risk.

The risk of septal heart defects in SSRI-exposed vs. unexposed infants was 0.9% vs. 0.5%; whereas the difference was statistically significant (adjusted OR, 1.99; 95% CI, 1.13-3.53), the absolute risk was small (number needed to harm, 246). Among the individual SSRIs, only citalopram and sertraline were associated with a significantly increased risk; however, previous data suggest that paroxetine is also associated with an increased risk of heart defects[12–13] and so it is likely that heart defects after first-trimester exposure to SSRIs is a class effect.[14] Of note, Pedersen *et al*,[11] observed that not all septal heart defects require treatment, and that some may even resolve spontaneously.

Finally, Pedersen *et al*,[11] found that the risk of a variety of other malformations, including CNS defects, neural tube defects, ocular defects, cardiac defects other than septal defects, cleft lip and cleft palate, gastrointestinal defects, urogenital defects, and other conditions did not differ significantly between SSRI-exposed and unexposed infants.

In a large study of 9849 infants with birth defects and 5860 infants without, Louik *et al*,[15] specifically examined the risks associated with craniosynostosis, omphalocele, and heart defects in relation to SSRI exposure. They found that first-trimester exposure to paroxetine or sertraline was associated with an increased risk of specific but uncommon congenital malformations such as omphalocele and cardiac defects.

In another large study of 9622 infants with major birth defects and 4092 control infants, Alwan *et al*,[16] identified first-trimester exposure to SSRIs in 2.4% of case infants and 2.1% of control infants; the difference was not significant. Overall, there were no significant associations between exposure to SSRIs during early pregnancy and congenital heart defects or most other categories or subcategories of birth defects. However, certain specific but rare birth defects were more common after SSRI exposure. These included anencephaly (OR, 2.4; 95% CI, 1.1-5.1), craniosynostosis (OR, 2.5; 95% CI, 1.5-4.0), and omphalocele (OR, 2.8; 95% CI, 1.3-5.7).

### B) Other pregnancy outcomes after exposure to SSRIs

1. True; 2. True; 3. True; 4. False; 5. False; 6. True; 7. True.

In a meta-analysis of nine studies with 2999 pregnancies, serotonin reuptake inhibitors were associated with a 70% increased risk of spontaneous abortion (OR, 1.7; 95% CI, 1.3-2.2).[17] Other studies have also demonstrated an increased risk of first trimester fetal waste associated with SSRI use.[10]

Lund *et al*,[9] described the effects of SSRIs on pregnancy outcomes in the prospective Aarhus Birth Cohort Study in Denmark. The sample comprised 1989-2006 data on 329 women who had used SSRIs during pregnancy, 4902 women with a positive psychiatric history (diagnosis mostly not recorded) but who had not used SSRIs during pregnancy, and 51,770 comparison women who had no psychiatric history and who had not used SSRIs during pregnancy. Women who received SSRIs and those with a psychiatric history but no SSRI use were older, of lower parity, and more often smokers than the comparison women. Statistical analysis adjusted for these and other confounding variables.

Lund *et al*,[9] found that the adjusted odds of preterm delivery, defined as birth before week 37 of gestation, were doubled in the SSRI-exposed women relative to women in the other two groups (crude risks, 8.8% vs. 5.0% vs. 4.9%, respectively). The mean gestational age, however, was just four to five days less in the SSRI-exposed pregnancies relative to pregnancies in the other two groups. Relative to infants in the other two groups, SSRI-exposed infants were four to seven times as likely to have a five-minute Apgar score of 7 or less (crude risks, 4.9% vs. 1.0% vs. 1.2%, respectively); and they were twice as likely to require neonatal ICU admission (crude risks, 16.4% vs. 9.0% vs. 7.4%, respectively). The risk of low birth weight (< 2.5 kg), the mean birth weight, and the mean head circumference did not differ significantly across infants in the three groups.

Persistent pulmonary hypertension in the newborn (PPHN) is associated with high morbidity and mortality; the incidence of this condition is about 0.1-0.2%. In a case-control study of 377 infants with PPHN matched with 836 controls, Chambers *et al*,[18] found that fetal exposure to SSRIs after week 20 of gestation was associated with a six-fold increase in the risk of PPHN (OR, 6.1; 95% CI, 2.2-16.8). Neither SSRI exposure before week 20 (OR, 0.3; 95% CI, 0.1-1.2) nor exposure to other antidepressants at any time during pregnancy (OR, 0.8; 95% CI, 0.2-2.7) significantly influenced the risk.

In a more recent study, Kallen and Olausson[19] examined 1997-2005 data on 831,324 births from the Swedish Medical Birth Register and collated these data with information about maternal exposure to drugs. Analyses were adjusted for confounders such as greater maternal age, primiparous status, greater BMI, and maternal smoking, all of which increase the risk of PPHN. Kallen and Olausson[19] found that maternal use of SSRIs during early pregnancy (n=7587) doubled the risk of PPHN (Relative Risk [RR], 2.4; 95% CI, 1.2-4.3). Furthermore, maternal use of SSRIs during late pregnancy (n=2350) trebled the risk of PPHN (RR, 3.6; 95% CI, 1.2-8.3).

There have been at least two other recent studies on the subject. Andrade *et al*,[20] matched 1104 infants exposed to SSRIs during the final trimester of pregnancy with 1104 controls; and Wichman *et al*,[21] compared 808 women who had used SSRIs or venlafaxine at some point during pregnancy with 24,406 control mothers. Neither study found an association between maternal use of SSRIs and PPHN. Both studies, however, were probably underpowered.

Why would SSRIs increase the risk of PPHN? It is possible that SSRI-related increase in serotonin levels in the fetal circulation could result in pulmonary vasoconstriction and proliferation of pulmonary smooth muscle, resulting in increased pulmonary resistance.

Finally, a mild to severe SSRI withdrawal syndrome may develop in about 30% of neonates after prolonged exposure to an SSRI during the last weeks of pregnancy. The syndrome comprises nonspecific symptoms such as irritability, crying, rapid breathing, poor feeding, and sleep disturbance. The withdrawal syndrome peaks in severity during the first two days after birth; in some cases, however, the peak may not occur until the fourth day of life.[22–24]

### C) SSRI-exposed vs. SSRI-unexposed pregnancies in depressed women

1. False.

The introduction to this article listed fetal and neonatal as well as maternal risks of leaving depression untreated during pregnancy; a logical conclusion, therefore, is that depression during pregnancy should not be left untreated. However, SSRIs are associated with definite fetal and neonatal risks, as discussed in the previous sections.

### So, which is the lesser of the two evils: treated depression or untreated depression?

The only way to resolve the issue is to compare pregnancies in which depression has been treated OR LEFT untreated. A challenge here is that observational data are bound to be biased: medication is likely to have been prescribed only to more severely depressed women who are perhaps predisposed to worse outcomes as a result of the greater severity of illness. Oberlander *et al*, got around this difficulty in a clever and illuminating study.

The sample comprised all live births (n=119,547) recorded during a 39-month period in a population health database in Canada. Depression was recorded in 14% of the mothers, and the incidence of prenatal SSRI exposure rose from 2.3% to 5.0% across the 39 months. There were three groups of interest: neonates born to depressed mothers who had used SSRIs during pregnancy (“doubly-exposed”; n=1451), neonates born to depressed mothers who had not used psychotropic drugs during pregnancy (“depression-exposed”; n=14,234), and neonates born to women who were neither depressed nor exposed to psychotropic medication during pregnancy (“doubly-unexposed”; n=92,192). To control for the severity of maternal illness, propensity score matching (based on a large number of socio-demographic and illness variables) was used to identify a comparison group of untreated depressed mothers (n=805) who were similar to the SSRI-exposed mothers (n=817) in the year preceding and during pregnancy.

In line with literature, Oberlander *et al.* found that on almost all outcome measures, depression-exposed neonates fared significantly worse than doubly-unexposed neonates. The differences, however, were very small. They also found that, on various indices, the depressed women who received SSRIs during pregnancy were more severely ill than the depressed women who did not receive SSRIs. Understandably, therefore, birth weight and gestational age were both significantly lower in doubly-exposed relative to depression-exposed and doubly-unexposed neonates. The difference, however, was again very small: about 30-50 g for birth weight, and about two to three days for gestational age. Relative to doubly-unexposed neonates, doubly-exposed neonates were also lighter for gestational age, and more likely to have been born through cesarean section. Finally, doubly-exposed neonates experienced more adverse outcomes than depression-exposed neonates; these outcomes were neonatal respiratory distress (13.9% vs. 7.8%), jaundice (9.4% vs. 7.5%), and feeding problems (3.9% vs. 2.4%). They also had longer hospital stays.

Importantly, when outcomes were compared between propensity-matched doubly-exposed and depression-exposed neonates, SSRI exposure was associated with a higher incidence of birth weight below the 10th percentile and a higher rate of neonatal respiratory distress. The two groups were otherwise similar in outcomes such as gestational age, birth weight, feeding problems, convulsions, and jaundice.

Propensity matching is not as good a research design as randomization because it is based on proxies of comparability, not true comparability. However, it is the best that is available in a situation in which randomized controlled trials will never be conducted.

A take-home message, therefore, is that milder depressions may be better managed through psychosocial interventions; however, the use of SSRIs during pregnancy can indeed be considered in moderate or severe depressions because the additional risks with SSRIs are not large. As always, all decisions should be individualized and made in consultation with the patient and her family.
